# Mitochondrial DNA haplogroup analysis in Saudi Arab patients with multiple sclerosis

**DOI:** 10.1371/journal.pone.0279237

**Published:** 2022-12-19

**Authors:** Ghada Al-Kafaji, Materah Salem Alwehaidah, Manahel Mahmood Alsabbagh, Maram A. Alharbi, Moiz Bakhiet

**Affiliations:** 1 Department of Molecular Medicine and Al-Jawhara Centre for Molecular Medicine, Genetics, and Inherited Disorders, College of Medicine and Medical Sciences, Arabian Gulf University, Manama, Kingdom of Bahrain; 2 Department of Medical Laboratory, Faculty of Allied Health, Kuwait University, Kuwait City, Kuwait; 3 College of Forensic Sciences, Naif Arab University for Security Sciences, Riyadh, Kingdom of Saudi Arabia; Imam Abdulrahman Bin Faisal University, SAUDI ARABIA

## Abstract

Previous studies have suggested that mitochondrial DNA (mtDNA) variants are associated with multiple sclerosis (MS), a complex neurodegenerative immune-mediated disease of the central nervous system. Since mtDNA is maternally inherited without recombination, specific mtDNA variants defining genetic background are associated with the susceptibility to human diseases. To assess the contribution of mtDNA haplogroups to the predisposition of MS in an Arab population, we analysed sequencing data of mitochondrial genomes from 47 native Saudi Arab individuals including 23 patients with relapsing-remitting MS (RRMS) and 24 healthy controls. All patients and controls could be classified into ten haplogroups. The European-specific haplogroup U was more prevalent in patients than in the controls (26.1% vs. 4.2%), whereas haplogroup T was only present in patients and haplogroups HV and N were only found in controls. Haplogroup U was significantly association with increased risk of MS (odds ratio = 6.26, p<0.05), although the association did not maintain significance after adjustment for multiple comparisons. Haplotype U was more prevalent in patients with younger age of onset (p = 0.006), but there was no relationship between haplotype U and disease severity, disease duration or EDSS and age-matched carriers and non-carriers of haplogroup U (p>0.05). Definition site of haplogroup U include the variant m.12308A>G in *MT-TL2* gene which was found to affect highly conserved position within the variable arm of tRNA^Leu(CUN)^ and thus may impact mitochondrial protein synthesis, and two other variants namely m.11467A>G in *MT-ND4* gene and m.12372G>A in *MT-ND5* gene which were previously linked with mitochondrial function. Despite the small number of subjects, which may limit the statistical power of the study, our results showed for the first time a possible contribution of haplogroup U to the predisposition to MS in an Arab population. These findings warrant further validation in a large cohort to distinguish a genuine effect specific to MS from a chance finding due to small sampling.

## Introduction

Multiple sclerosis (MS) is a complex immune-mediated neurodegenerative disease of the central nervous system (CNS) and the most common cause of neurological disability in young adults [1, 2]. The disease usually occurs in females two to three times more frequently than males [3]. In MS, the immune system attacks the myelinated axons in the brain and spinal cord leading to inflammatory demyelination, and diffuse neurodegeneration in both grey and white matter of the brain and spinal cord [4, 5]. MS is categorized into four types which are considered important for prognosis and treatment decisions: relapsing-remitting MS (RRMS), primary progressive MS (PPMS), secondary progressive MS (SPMS), and progressive relapsing MS (PRMS). RRMS is the most common type, which occurs in approximately 87% of cases and characterized by unpredictable acute attacks followed by periods of remission [5]. Based on a recent epidemiology survey, the global prevalence of MS has increased to 2.8 million with Europe having the highest reported incidence rate, followed by America [6]. In addition, the incidence of MS has increased in the Arab Gulf countries, including United Arab Emirates, Saudi Arabia, Qatar, Oman, Bahrain and Kuwait [7, 8].

The exact cause of MS is unknown, but it is believed that multiple factors are involved in its etiology including environmental factors and DNA defects in nuclear and mitochondrial genomes [9, 10]. The hypothesis that mitochondrial genes may be implicated in the susceptibility to MS was originated based on the observations of maternal transmission in familiar MS cases [11, 12] and the occurrence of neurological features including inflammatory demyelination compatible with a diagnosis of MS in patients with Leber’s hereditary optical neuropathy (LHON), a mitochondrial disorder caused by mtDNA mutations [13]. This hypothesis was also supported by the detection of mtDNA mutations and common polymorphisms in patients with MS [14–16], which play an important role in the pathogenicity and susceptibility of MS. Because the mitochondrion provide most of the energy for the cell, they preferentially affect tissues with high-energy such as brain and thus dysfunction of the mitochondria and the resulting depleted energy supply contribute to the progression of MS [17, 18]. Human mtDNA is circular, double-stranded molecule and contains 37 genes coding for 13 polypeptides of the respiratory chain complexes that are required for oxidative phosphorylation (OXPHOS) as well as 22 transfer RNAs (tRNAs) which translate essential subunits of the respiratory chain complexes and 2 ribosomal RNAs (rRNAs), which direct the catalytic steps of protein synthesis [19]. Moreover, the mtDNA contains a D-loop region, the only non-coding region that is considered to be important in mtDNA expression and replication [19]. The mtDNA shows a higher mutation rate compared with nuclear DNA (nDNA), likely due to less efficient DNA repair capacity, absence of protective histone, and high ROS exposure [20]. Since mtDNA is maternally inherited without recombination [21], specific mtDNA variants defining some genetic backgrounds are associated with the susceptibility to human diseases [22]. Haplogroups are collections of similar haplotypes defined by unique sets of variants in mtDNA inherited from a common ancestor and accumulated by a discrete maternal lineage [23]. In the mitochondrial phylogenetic tree, haplogroups are identified as major branch points known as major mitochondrial haplogroups which allowed human populations to be categorized into various mtDNA haplogroups [23]. However, the sets and frequencies of these haplogroups differ between populations and have been used in association studies to assess the role of mtDNA variants in various complex diseases. In certain populations, these haplogroups confer risk or resistance against certain disease. For instance, in Russian Tatar population polymorphisms associated with mtDNA haplogroup H increased the risk of Parkinson’s disease (PD), whereas those associated with haplogroup UK cluster were protective [24]. On the contrary, a reduced risk of Alzheimer disease (AD) was reported for variants in haplogroup H [25]. In MS, some studies showed an association of haplogroup U in Caucasians [26] but not in a Russian patients [27]. Studies also reported a consistent association of haplogroup J with the risk of MS in Russian [27, 28], Persian [29] and Bulgarian [30] patients, while others failed to find any association of haplogroup J with MS in Basque patients [31].

We recently sequenced the entire mitochondrial genome from unrelated native Saudi Arab individuals including patients with RRMS and healthy controls using next-generation sequencing (NGS), and identified unique and common mtDNA mutations/variants [32]. In this study, we assessed the hypothesis that mtDNA haplogroup and polymorphisms maybe an important factor in the susceptibility of MS.

## Methods

### Subjects

A total of 47 unrelated native Arab individuals from Saudi Arabia including 23 patients with relapsing-remitting form of MS (RRMS) who had been referred to the Neurology Clinic at King Khalid Hospital, King Saud University, Saudi Arabia and 24 healthy controls were enrolled in the study. The RRMS patients were diagnosed according to the revised McDonald criteria [33], in which at least two previous relapses in the CNS regions were confirmed by a neurological examination, magnetic resonance imaging (MRI) scans, and electrophysiological studies. The control subjects who had no neurological conditions or history of autoimmune and inflammatory disease were King Khalid hospital blood donors. Age at the onset of the first symptoms, gender, disease duration of symptoms, clinical disability according to Kurtzke Expanded Disability Status Scale (EDSS), and medications were reported for all patients. Age, gender and other data including blood pressure and body mass index (BMI) were also recorded for both patients and controls. All of the patients and controls were informed on the aim of the study and signed written informed consents for their participation. Ethical approval to conduct the study was obtained from the Scientific and Ethics Committee in King Saud University, College of Medicine (Saudi Arabia), and the Medical Research and Ethics Committee in the College of Medicine and Medical Sciences, Arabian Gulf University (Bahrain). All methods were carried out as per Ethical Guidelines of the above mentioned Institutional Ethics Committees.

### Clinical samples and DNA extraction

Blood samples were collected in 5 ml ethylenediaminetetraacetic acid (EDTA) tubes from all the subjects. Genomic DNA was extracted from peripheral whole blood using QIAMP DSP DNA kit (Qiagen, Hilden, Germany) according to the recommendations of the manufacturer and as previously described [16, 32]. All DNA samples were quantified and checked for purity using the NanoDrop ND-1000 ultraviolet-visible light spectrophotometer (Thermo Fisher Scientific, Inc.).

### Mitochondrial genome sequencing and analysis

The entire mitochondria genome was sequenced using HiSeq X NGS. The generated data sets of mtDNA sequences are available in NCBI [32]. The mtDNA was amplified with Long-range PCR (Qiagen Long range PCR kit, Cat# 206403) with two sets of primers: L644 (GACGGGCTCACATCACCCCATAA) and H8982 (GCGTACGGCCAGGGCTATTGGT) for fragment 1 and L8789 (GCCACAACTAACCTCCTCGGACTCCT) and H877 (GGTGGCTGGCACGAAATTGACC) for fragment 2. PCR products were subsequently purified and quantified before proceeding to DNA library preparation for sequencing according to HiSeq X Illumina protocol. Based on the quality of FASTQ files, sequence reads were trimmed to only high quality sequence for further analysis, and the paired end reads were aligned to the reference human genome GRCh37/hg19 available from UCSC genome browser. Identification of variants including single nucleotide polymorphisms and short Indels as well as quality of variant calls were done using GATK-lite program. The Revised Cambridge Reference Sequence (rCRS), (NCBI Reference Sequence: NC_012920) was used as mitochondrial reference genome (http://www.mitomap.org/MITOMAP/Human/Mitoseq). All mtDNA variants were submitted to the GenBank database [32].

### mtDNA haplogroup classification and phylogenetic analysis

The mtDNA haplogroups were determined using HaploGrep 2 [34] under default setting according to the criteria of MITOMAP. Classification of mtDNA haplogroups was done with PhyloTree, Build 17, which comprises nearly 5500 haplogroups [35]. All mtDNA variants were entered for the haplogroup determination, and each individual’s haplogroup was determined based on PhyloTree build 17. We only included haplogroups with a quality score above 80% and with a frequency above 1% [35]. Then phylogenetic trees were constructed which showed the distribution of the haplogroups. To evaluate mtDNA variants of the particular branches of each haplogroup, the location of protein-encoded, RNA-encoded and control region variants were included in phylogenetic analysis and construction of haplogroups.

### Conservation assessment

A phylogenetic approach was performed to determine the evolutionary conservation of the mitochondrial tRNA (MT-tRNA) variant. A total of 16 vertebrate mtDNA sequences from the NCBI database were used in the interspecific analysis and alignment was retrieved from http://mamit-trna.u-strasbg.fr/. The conservation index (CI) was calculated by comparing the human nucleotide variants with other 15 vertebrates. A CI of ≥75% was considered as having functional potential [36].

### Structural analysis

The published secondary structures of the MT-tRNAs were used to define the stem and loop structure [37, 38]. The FASTA format of the sequences was submitted to web servers for tRNA secondary structures prediction (Mamit-tRNA http://mamit-trna.u-strasbg.fr/).

### Statistical analysis

Analysis of data was performed using the statistical package SPSS (version 23; IBM Corp., Armonk, NY, USA). Comparison of demographic and clinical parameters between cases and controls was done using a Chi-square test for categorical variables or equivalent non-parametric Wilcoxon signed-rank test and Mann-Whitney test. For association analysis, sub-haplogroups were first assigned to their respective major haplogroups and Fisher’s exact test was used to assess the differences in haplogroup frequencies between cases and controls. The risk of association was estimated by computing the odds ratio (OR) and 95% confidence interval (CI). A two sided p-value below 0.05 was considered to be statistically significant. Multiple comparisons correction was performed using the Bonferroni correction. Since we examined ten mtDNA haplogroups, Bonferroni correction of a p-value of less than 0.005 (0.05/10) was considered significant.

## Results

### Characteristics of study subjects

Table 1 shows the characteristics of study subjects. Data are presented as number, percentage or mean ± standard deviation (SD). The RRMS patient group consisted of 23 subjects with a mean age of 28 years (± 7.5) and ranged from 18–44 years. The control group consisted of 24 subjects with a mean age 31 (± 7.5) and ranged from 22–52 years. Out of 23 patients, 18 (78.3%) were females and 5 (21.7%) were males (female to male ratio was ~ 3:1). Likewise, in the control group 19 (79.2%) were females and 5 (20.8%) were males (female to male ratio was ~ 3:1). No significant differences were found between the two groups in terms of mean age, mean BMI and mean blood pressure (p>0.05). The patients were under the following treatment: Avonex (n = 3), Betaferon (n = 8), Glienya (n = 3), Rebif (n = 5) and Tysabri (n = 4).

**Table 1:** Characteristics of study subjects.

	RRMS patients	Healthy controls	p-value
Number of subjects	23	24	
Age (mean ± SD, range)	28 ± 7.5, 18–44	31 ± 7.5, 22–52	0.12
Female (n, %)	18, 78.3	19, 79.2	1
Male (n, %)	5, 21.7	5, 20.8	1
BMI (mean ± SD)	27 ± 6.2	29 ± 5.5	0.15
Blood pressure (mean ± SD)	88.9 ± 10.9	89.8 ± 22.5	0.86
Disease duration (mean ± SD, range)	5.3 ± 4.0, 1–15		
EDSS (mean ± SD, range)	3.9 ± 1.4, 2–6.5		
Presenting symptoms (n, %)			
Numbness	23, 100		
Visual problem	20, 86.9		
Muscle spasticity	18, 78.3		
Balance problem	13, 56.5		
Slurred speech	10, 43.5		
Psychiatric condition	14, 60.7		
Medication			
Avonex	3		
Betaferon	8		
Glienya	3		
Rebif	5		
Tysabri	4		

Data are presented as number, percentage (%) or mean ± standard deviation (SD). RRMS, relapse-remitting multiple sclerosis; EDSS, Expanded Disability Status Scale. p-value significant threshold = 0.05.

### Distribution of mitochondrial haplogroups among patients and controls

**Fig 1:**
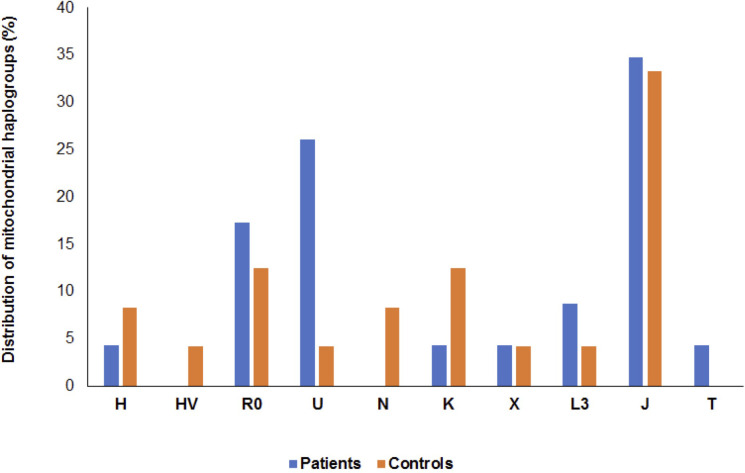
Distribution of mitochondrial haplogroups among patients and controls. A total of ten haplogroups (H, HV, R0, U, N, K, X, L3, J and T) were observed in patients and controls that showed differences in their distribution among the two groups.

From the mtDNA sequencing data, distribution of mitochondrial haplogroups was analyzed in 23 patients with RRMS and 24 healthy control subjects. A total of ten haplogroups were observed in patients and controls that showed differences in distribution among the two groups. These include haplogroups H, HV, R0, U, N, K, X, L3, J and T (Fig 1). A higher occurrence of haplogroup U was observed in patients compared to controls. Haplogroup T was only found in the patient group, whereas haplogroups HV and N were only found in the control group. Phylogenetic tree based on completed mtDNA sequences of patients and controls are shown in S1 and S2 Figs respectively.

### Association of mitochondrial haplogroups with the susceptibility to MS

We next analyzed the association of mitochondrial haplogroups with the susceptibility to MS using the Fischer exact test and Bonferroni correction test. The OR and 95% CI were reported. As shown in Table 2, the frequency of haplogroup U in the patients was higher compared with that of the controls (26.1% vs. 4.2%). Statistical analysis revealed a significant association of haplogroup U with the increased risk of MS (OR = 6.261, 95% CI = 0.82–48.1, p = 0.042). This was confirmed with linear-by-linear association test which showed a statistical significant result for the association of haplogroup U with MS (p = 0.037). However, the association did not survive Bonferroni multiple testing. For all other haplogroups, no significant association with MS was observed (p>0.05).

**Table 2:** Association of mitochondrial haplogroups with the susceptibility to MS.

	RRMS patients (n = 23)	Healthy controls (n = 24)			
Haplogroup	No (frequency %)	No (frequency %)	OR	95% CI	*p-value
H	1 (4.3)	2 (8.3)	0.52	0.05–5.37	1
HV	0 (0)	1 (4.2)	1.043	0.96–1.13	1
R0	4 (17.3)	3 (12.5)	1.391	0.35–5.55	0.70
U	6 (26.1)	1 (4.2)	6.26	0.82–48.1	0.042
N	0 (0)	2 (8.3)	1.09	0.97–1.23	0.49
K	1 (4.3)	3 (12.5)	0.348	0.39–3.017	0.61
X	1 (4.3)	1 (4.2)	1.043	0.69–15.72	1
L3	2 (8.7)	1 (4.2)	1.043	0.16–6.80	1
J	8 (34.8)	8 (33.3)	1.043	0.47–2.31	1
T	1 (4.3)	0 (0)	0.957	0.87–1.04	0.49

RRMS, relapse-remitting multiple sclerosis; OR, odds ratio; CI, confidence interval.

*p-value significant threshold = 0.05, Bonferroni adjusted significance threshold = 0.005.

### Relationship between haplogroup U and clinical parameters

Since haplogroup U was most prevalent in patients with MS and showed an association with MS by Fischer exact test and linear-by-linear test, we sought to determine the relationship between this haplogroup and clinical parameters. We compared the age of onset of the first symptoms, disability scores (as evaluated by EDSS) and duration of symptoms in patients with haplogroup U and non-haplogroup U patients. As shown in Table 3, 17.3% of patients with haplogroup U were younger in age of onset (mean age 19.5 ± SD 1.3) compared to 73.9% of non-haplogroup U patients (mean age 30.4 ± SD 6.79), (p = 0.006). However, no significant relationship was found between haplogroup U and EDSS (p = 0.59) or disease duration (p = 0.23).

**Table 3:** Relationship between haplogroup U and clinical parameters in RRMS patients.

	Haplogroup U		Non-haplogroup U		
	Percentage (%)	Mean ± SD	Percentage (%)	Mean ± SD	p-value
Age of disease onset	17.3	19.5 ± 1.29	73.9	30.4 ± 6.79	0.006
EDSS	26.1	4.0 ± 1.56	73.9	4.4 ± 1.74	0.59
Disease duration	26.1	3 ± 2.04	73.9	5 ± 3.42	0.23

Data are presented as percentage (%) and mean ± standard deviation (SD). RRMS, relapse-remitting multiple sclerosis; EDSS, Expanded Disability Status Scale. p-value significant threshold = 0.05.

To consider additional factors in the association between haplogroup U and clinical characteristics, we compared EDSS between age-matched carriers and non-carriers of haplogroup U. As shown in Table 4, no statistical significant result was found between carriers of haplogroup U with a mean age 26.8 ± SD 4.45 and non-haplogroup U carriers with a mean mean age 26.8 ± SD 3.0 (p = 0.10).

**Table 4:** Relationship between EDSS and age-matched carriers and non-carriers of haplogroup U.

	Haplogroup U	Non-haplogroup U	
	Mean ± SD	Mean ± SD	p-value
Age	26.8 ± 4.45	26.8 ± 3.0	
EDSS	4.45 ± 0.97	3.0 ± 1.3	0.10

Data are presented as percentage mean ± standard deviation (SD). EDSS, Expanded Disability Status Scale. p-value significant threshold = 0.05.

### Variants determining mitochondrial haplogroup U

**Fig 2:**
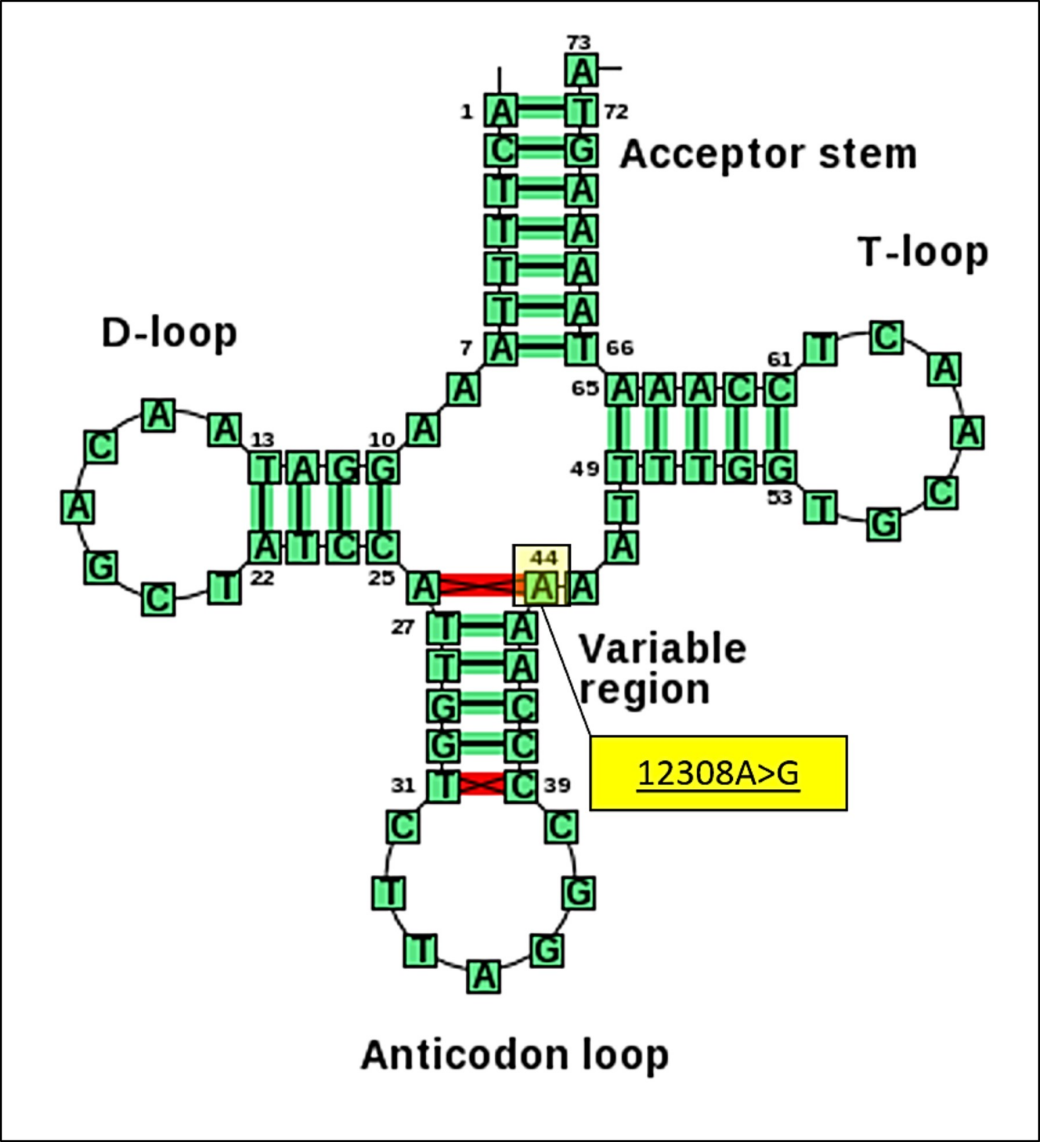
Secondary structure prediction of tRNA^Leu(CUN)^ by the web servers for tRNA secondary structures (Mamit-tRNA http://mamit-trna.u-strasbg.fr/). The m.12308A>G variant created a novel Watson-Crick base-pairing (A25-T43) in the variable region of mitochondrial tRNA^Leu(CUN)^.

In subsequent analysis, we identified variants determining mitochondrial haplogroup U, which was found to be more prevalent in patients with MS. As shown in Table 5, definition site of haplogroup U include the variant m.12308A>G in *MT-TL2* gene and two variants in protein-coding genes namely m.11467A>G (referred as rs2853493 polymorphism) in *MT-ND4* gene and m.12372G>A (referred as rs2853499 polymorphism) in *MT-ND5* gene. These three variants were more prevalent in patients than in controls (26.1% vs 4.2%, p<0.05). Since m.11467A>G of *MT-ND4* gene and m.12372G>A of *MT-ND5* gene are silent variants, we focused on m.12308A>G variant in *MT-TL2* gene and assessed its functional effect. First a phylogenetic approach was used to see the evolutionary conservation of m.12308A>G and alignment was retrieved from http://mamit-trna.u-strasbg.fr/. The results showed that the A nucleotide at position 44 *MT-TL2* gene encoding tRNA^Leu(CUN)^ is highly conserved between various species with a CI of 100% (S3 Fig). Then, the secondary structure of the mt-tRNA was used to localize the variant with either a stem or a loop to assess whether the base change altered the classic Watson-Crick base-pairing. As shown in Fig 2, m.12308A>G created a novel Watson-Crick base-pairing (A25-T43) at the variable arm of tRNA^Leu(CUN)^.

**Table 5:** mtDNA variants determining haplogroup U in this study.

Gene	Nucleotide change	Amino acid change	Type of variant	Variant ID
*MT-TL2*, tRNA^Leu(CUN)^	m.12308A>G	-	Substitution	NP
*MT-ND4*	m.11467A>G	p.Leu236 (=)	Silent	rs2853493
*MT-ND5*	m.12372G>A	p.Leu12 (=)	Silent	rs2853499

## Discussion

MS is a multifactorial disease, the pathogenesis involves both environmental genetic factors. Of these variants in the mitochondrial genome (mtDNA) contribute to the pathogenesis and risk [10, 14–16]. Recent studies have reported that mtDNA background affects the expression of human disease such as PD and AD [24, 25]. MS has also been reported to be modulated by mtDNA haplogroups [26–31]. Most of these reports have shown population specific associations of diseases with particular haplogroups. In this study, we compared the whole mtDNA sequences from 47 unrelated native Arab individuals from Saudi Arabia including 23 RRMS patients and 24 healthy controls to assess the prevalence of and role of mitochondrial haplogroups in MS in this Arab cohort. Ten haplogroups namely H, HV, R0, U, N, K, X, L3, J and T were observed in patients and controls that showed differences in distribution among the two groups (Fig 1). A comparison of these haplogroups revealed a higher prevalence of haplogroup U in patients compared to controls. Moreover, haplogroup T was only detected in the patient group, whereas haplogroups HV and N were only found in the control group. Association analysis (Table 2) revealed a significant association of haplogroup U with MS using the Fischer exact test and linear-by-linear test. However, the association did not maintain significance after adjustment for multiple comparisons, and thus the possibility of it being a false positive result cannot be eliminated. Haplogroup U is a major haplogroup in European populations. In a large study involving Caucasian patients with MS and controls, haplogroup U was reported as a risk factor in MS [26]. On the contrary, it was not associated with MS in a Russian population [27]. Haplogroup U has been implicated in various human pathological conditions such as neurodegenerative diseases [39] and psychiatric disorders [40].

Moreover, we did not find a significant difference in the frequency of haplogroup K between patients and controls. Similar to our results, no association between haplotype K and MS was reported in European [41] or Russian [27] populations. However, specific variants in haplogroup K were found to contribute moderately to MS susceptibility in Caucasians [42]. We also did not observe a significant difference in the frequency of haplogroup J between patients and controls. An earlier study by Reynier et al, [43] found haplogroup J only in MS patients with optic neuritis (ON) but not in MS patients without visual symptoms, and suggested that this haplogroup might constitute a risk factor for ON occurrence when it is coincidentally associated with MS, but not as a risk factor for developing MS. On the other hand, other studies have revealed a moderate effect of haplogroup J with MS susceptibility in Caucasians [42] and a significant association with MS in Russian [27, 28], Persian [29] and Bulgarian [30] patients. Results from this study also revealed the presence of haplogroup T in MS patients only. Contrary to our finding, Tranah et al, [44] showed an association of haplogroup T with the risk of MS in non-Hispanic whites.

The discrepancy between our data and other studies may be due to racial and geographical differences in haplogroup structure [45] together with nuclear background or environmental risk factors [46, 47]. It seems likely that each haplogroup, with its different set of variants can have unique association with altered MS risk in different populations. Consequently, adjusting for confounders is very important in terms of false positive results, which could impact the association of mtDNA haplogroups with MS.

Since haplogroup U was more prevalent in MS patients in our study and was previously reported as a risk factor of MS [26], we wanted to determine the relationship between this haplogroup and clinical parameters in MS patients (Table 3). Our results showed that haplogroup U was more prevalent in patients with younger age of disease onset compared to non-haplogroup U patients. The correlation of haplogroup U with the age of disease onset may suggest that patients with this haplogroup may manifest the disease at younger ages. While anticipation is most often seen with certain genetic disorders of the nervous system such as Huntington disease and Fragile X syndrome, an anticipation of the age at onset has been reported in younger generations of patients with familial MS [48, 49], which could be attributed to environmental changes [49]. On the other hand, no relationship was found between haplogroup U and EDSS or disease duration (Table 3). Moreover, when we compared EDSS between age-matched carriers and non-carriers of haplogroup U (Table 4), no significant relationship was observed. Nevertheless, additional factors should be considered when analysing the association of genotype with clinical variables such as the relationship between haplogroup U and disease progression.

In our analysis, haplogroup U is defined by three variants namely m.12308A>G, m.11467A>G and m.12372G>A (Table 5 and S1 and S2 Figs). m.12308A>G is located in the *MT-TL2* gene, which codes for the most represented amino acids in the mitochondrial respiratory chain and plays a key role the OXPHOS subunit genes. Based on our phylogenetic conservation analysis (http://mamit-trna.u-strasbg.fr/), the A nucleotide at position 44 of *MT-TL2* gene was found to be highly conserved between various species with a CI of 100% (S3 Fig). Moreover, based on the secondary structure of the MT-tRNA, per the Mamit-tRNA database (http://mamit-tRNA.u-strasbg.fr) (Fig 2), m.12308A>G was found to affect highly conserved position and created a novel Watson-Crick base-pairing (A25-T43) at the variable arm of tRNA^Leu(CUN)^ encoded by *MT-TL2* gene. The variable arm of tRNA is located between the anticodon and the T arms, and functions as a stabilizer of the tertiary structure and also in the specific recognition of the aminoacyl-tRNA synthetase (ARS), also called tRNA-ligase, an enzyme that attaches the appropriate amino acid onto its corresponding tRNA during translation. High level of accuracy is required by the aminoacylation reaction to provide a critical checkpoint for translational quality control, and mistranslation occurs when an amino acid is attached to the wrong tRNA and subsequently is misplaced in a nascent protein [50]. The necessity for proper MT-tRNA function is evident from numerous human diseases associated with mutations or incorrectly processed tRNAs, particularly in high-energy consuming tissues that are mainly affected by mitochondrial defects. Therefore, normal Watson–Crick base-pairing is crucial for maintaining the proper structure and function of MT-tRNAs and any modifications in MT-tRNAs may lead to low accuracy and efficiency of mitochondrial protein synthesis. In fact, modifications of tRNA caused by mutations are linked to numerous mitochondrial and neurological diseases [37, 51]. By literature searching, we also noticed that m.12308A>G of tRNA^Leu(CUN)^ had been reported to be associated with mitochondrial encephalomyopathies [52] and syndromes of Wolfram and chronic progressive external ophthalmoplegia [53]. It was also reported to be associated with increased ROS production and considered a risk factor for many diseases such as severe knee osteoarthritis [54], stroke [55], breast cancer [56] and AD in men [39]. According to these observations, it might be possible to speculate the molecular mechanism underlying haplogroup U-related m.12308A>G in MS. m.12308A>G alters the secondary structure and affectes the steady-state levels of tRNA^Leu(CUN)^, and may subsequently lead to the failure in tRNA aminoacylation and mitochondrial protein synthesis. This may cause a decline in ATP and an increase in ROS production and eventually mitochondrial dysfunction, which is implicated in the pathogenesis of MS [10].

The other two variants of haplogroup U are m.11467A>G and m.12372G>A, located in the *MT-ND4* and *MT-ND5* genes respectively. ND4 and ND5 are subunits of NADH dehydrogenase complex I, the largest multisubunit enzyme complex in the electron transport chain with a key role of transfer electrons from matrix NADH to ubiquinone. Complex I also plays an important role in ROS generation during cellular activity. However, impaired complex I function can lead to enhanced ROS generation and oxidative stress [57, 58], which are implicated in mitochondrial dysfunction and neuronal degeneration in MS [10]. In fact, m.11467A>G was previously reported in Egyptian patients with mitochondrial disease and optic atrophy [59]. Whereas, m.12372G>A was found in patients with LHON [60], a maternally inherited mitochondrial blinding disorder caused by mtDNA mutations and known to be associated with MS [13]. Moreover, both variants were reported in Creutzfeldt–Jakob disease, a degenerative brain disorder [61] and in patients with mitochondrial encephalomyopathies [52]. In addition, previous studies have identified some of the functional effects of these two variants. Both of them have been linked with pH alteration in brain [40], and may lead to reduce coupling due to loosing excess mitochondrial oxidation and decreased H+ ion gradients in the outer membrane [61]. Therefore, these two variants may relate to partial uncoupling of OXPHOS and defects in energy metabolism due to a deficiency in ATP synthesis in individuals derived from haplogroup U lineage.

While the relationship of haplogroup U with MS might be driven by the variants in its background, other non-haplogroup-associated variants [32], and genetic susceptibility factors such as haplotype HLA-DRB1*15:01~HLA-DQ*06:01 [62, 63], as well as nuclear genes and environmental factors involved in the etiology of MS may also play a role in predisposing individuals to the risk of MS.

Our study was limited by small sample size and additional extensive studies should be performed in order to detect even small associations between mtDNA haplogroups and MS and to distinguish a genuine effect specific to MS from a chance finding due to small sampling. Moreover, further work is needed to better understand the functional role of haplogroup U related polymorphisms and their interactions with other factors that affect MS.

## Conclusions

Our results showed for the first time that mtDNA background could influence the susceptibility to MS in an Arab population. Haplogroup U-related variants, which are linked to impaired mitochondrial function, may be inherited factors for MS in Saudi Arab patients. Further studies are needed to verify this conclusion.

## Supporting information

S1 Fig Phylogenetic tree based on complete mtDNA sequencing from 23 RRM patients. The haplogroups and corresponding variants are represented. The blue coloring represents local private mutations while the red coloring represents global private mutations.
(PDF)

S2 Fig Phylogenetic tree based on complete mtDNA sequencing from 24 healthy controls. The haplogroups and corresponding variants are represented. The blue coloring represents local private mutations while the red coloring represents global private mutations.
(PDF)

S3 Fig Sequence alignment of *MT-TL2* gene for tRNA^Leu(CUN)^ in different species. Alignment was retrieved from http://mamit-trna.u-strasbg.fr/. The arrow indicates the conservation of the A nucleotide throughout species (yellow highlight). The conservation index (CI) was 100% in all 16 organisms.
(PDF)
