# Supplementary figures and images for: Mitochondrial DNA haplogroup analysis in Saudi Arab patients with multiple sclerosis
Source: PLoS One. 2022 Dec 19;17(12):e0279237. doi: 10.1371/journal.pone.0279237 (PMC9762579; doi:10.1371/journal.pone.0279237)

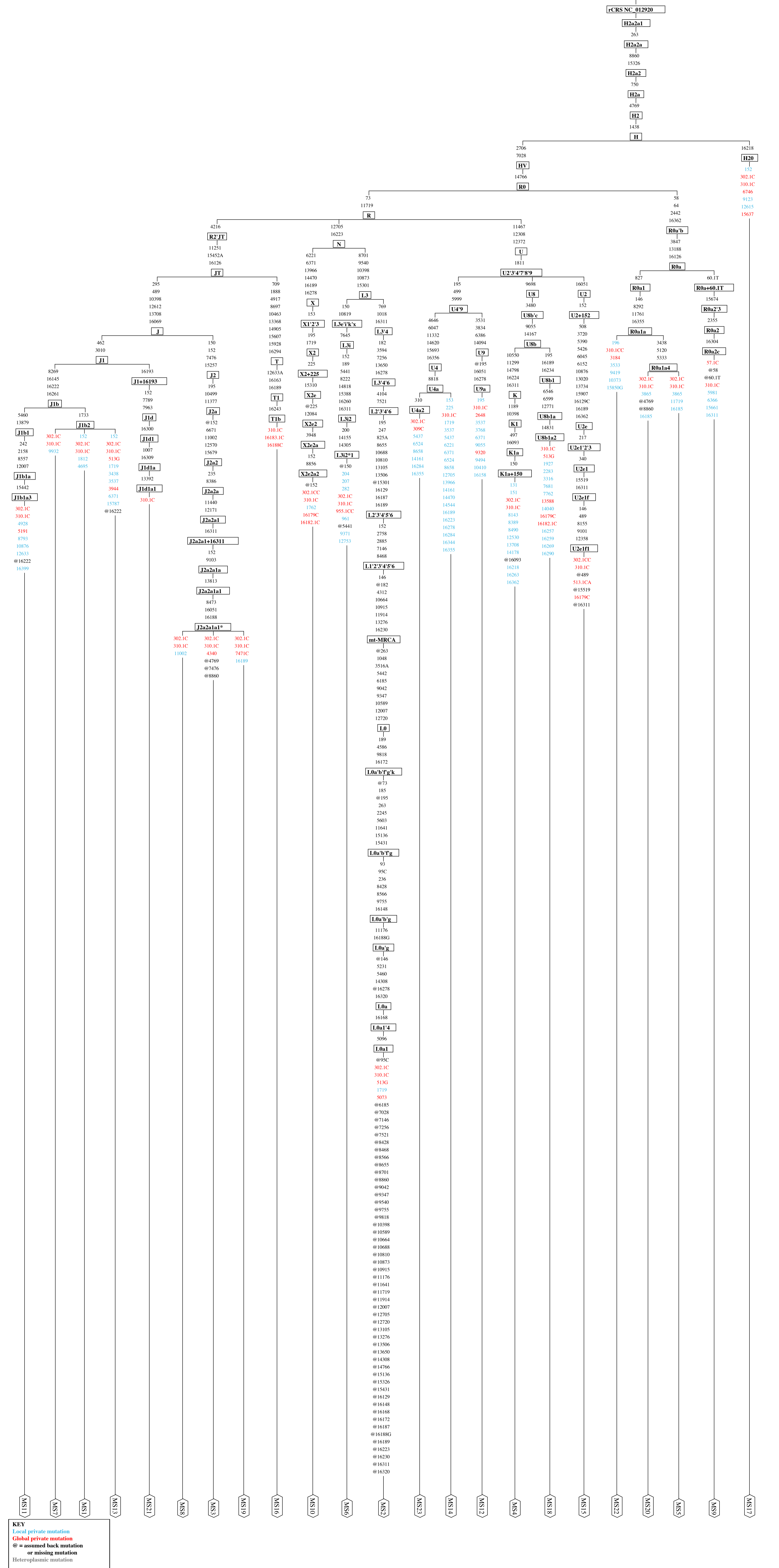

Supplement: S1 Fig — The haplogroups and corresponding variants are represented. The blue coloring represents local private mutations while the red coloring represents global private mutations. (PDF) [file pone.0279237.s001.pdf]
