# Supplementary material for: Mitochondrial DNA haplogroup analysis in Saudi Arab patients with multiple sclerosis
Source: PLoS One. 2022 Dec 19;17(12):e0279237. doi: 10.1371/journal.pone.0279237 (PMC9762579; doi:10.1371/journal.pone.0279237)
Supplement: S3 Fig — Alignment was retrieved from http://mamit-trna.u-strasbg.fr/. The arrow indicates the conservation of the A nucleotide throughout species (yellow highlight). The conservation index (CI) was 100% in all 16 organisms. (PDF) [file pone.0279237.s003.pdf]

12308

Organism	Acc-stem		D-stem	D-loop	D-stem		Ac-stem	Anticd-loop	Ac-stem	V-region	T-stem	T-loop	T-stem	Acc-stem	
	1	8	10		22	26	27	32	39	44	49		61	66	73
Bos taurus	ACTTTTA	AA	GGAT	AGTAGTTT	ATCC	G	TTGGT	CTTAGGA	ACCAA	AAA	ATTGG	TGCAACT	CCAAA	TAAAAGT	A
Cebus albifrons	GCTTTTA	AA	GGAT	AGTAGTT	ATCC	A	TTGGT	CTTAGGA	GCCAA	AAAT	ATTGG	TGCAACT	CCAAA	TAAAAGC	A
Gorilla gorilla	ACTTTTA	AA	GGAT	AACAGCT	ATCC	A	TTGGT	CTTAGGA	CCCAA	AAAT	TTTGG	TGCAACT	CCAAA	TAAAAGT	A
Homo sapiens	ACTTTTA	AA	GGAT	AACAGCT	ATCC	A	TTGGT	CTTAGGC	CCCAA	AAAT	TTTGG	TGCAACT	CCAAA	TAAAAGT	A
Hylobates lar	ACTTTTA	AA	GGAT	AACAGCT	ATCC	A	TTGGT	CTTAGGA	CCCAA	AAAT	TTTGG	TGCAACT	CCAAA	TAAAAGT	A
Lemur catta	ACTTTTA	AA	GGAT	AGAAGTA	ATCC	A	TTGGC	CTTAGGA	GCCAA	AAA	ATTGG	TGCAACT	CCAAA	TAAAAGT	A
Macaca mulatta	ACTTTTA	AA	GGAT	AACAGCT	ATCC	A	TTGAC	CTTAGGA	GTCAA	AAAC	ATTGG	TGCAACT	CCAAA	TAAAAGT	A
Macaca sylvanus	ACTTTTA	AA	GGAT	AACAGCT	ATCC	A	TTGGC	CTTAGGA	GTCAA	AAAT	ATTGG	TGCAACT	CCAAA	TAAAAGT	A
Mus musculus	ACTTTTA	TA	GGAT	AATAGTA	ATCC	A	TTGGT	CTTAGGA	ACCAA	AAAC	CTTGG	TGCAAAT	CCAAA	TAAAAGT	A
Nycticebus coucang	ACTTTTA	AA	GGAT	GGGAGCC	ATCC	G	TTGGT	CTTAGGA	GCCAA	AAA	ATTGG	TGCAACT	CCAAA	TAAAAGT	A
Pan paniscus	ACTTTTA	AA	GGAT	AACAGCC	ATCC	G	TTGGT	CTTAGGC	CCCAA	AAAT	TTTGG	TGCAACT	CCAAA	TAAAAGT	A
Pan troglodytes	ACTTTTA	AA	GGAT	AACAGTT	ATCC	A	TTGGT	CTTAGGC	CCCAA	AAAT	TTTGG	TGCAACT	CCAAA	TAAAAGT	A
Papio hamadryas	ACTTTTA	AA	GGAT	AACAGCT	ATCC	A	TTGGT	CTTAGGA	ACCAA	AAAC	ATTGG	TGCAACT	CCAAA	TAAAAGT	A
Pongo pygmaeus	ACTTTTA	AA	GGAT	AACAGCT	ATCC	C	TTGGT	CTTAGGA	CCCAA	AAAT	TTTGG	TGCAACT	CCAAA	TAAAAGT	A
Pongo pygmaeus abelii	ACTTTTA	AA	GGAT	AACAGCT	ATCC	C	TTGGT	CTTAGGA	CCCAA	AAAT	TTTGG	TGCAACT	CCAAA	TAAAAGT	A
Tarsius bancanus	ACTTTTA	AA	GGAT	AGAAGTA	ATCC	A	TCGGC	CTTAGGA	GCCGA	AAA	ATTGG	TGCAACT	CCAAA	TAAAAGT	A
